# Drinking Water

**Published:** 1873-07

**Authors:** 


### DRINKING WATER.

The longer one puts off drinking water in the morning, especially in summer, the less will he require during the day; if much is drank in the forenoon the thirst often increases and a very unpleasant fulness is observed, in addition to a metallic taste in the mouth.

The less water a man drinks the better for him, beyond a moderate amount. The more water a man drinks the more strength he has to expend in getting rid of it, for all the fluids taken into the system must be carried out—and, as there is but little nourishment in water, tea, coffee, beer and the like, more strength is expended in conveying them out of the system than they impart to the system. The more a man drinks the more he must perspire, either by the lungs or through the skin ; the more he perspires the more carbon is taken from the system, but this carbon is necessary for nutrition, hence the less a man is nourished the less strength he has.

The more liquids used the greater must be the amount of urination, but this detracts a proportional amount of albumen from the system, and it is the albumen in the food which strengthens us ; hence drinking water largely diminishes the strength in two ways, and yet many are under the impression that the more water swallowed the more thoroughly is the system "washed out ;" hence it follows that the less we drink at meals the better for us. If the amount was limited to a single cup of hot tea or hot milk and water at each meal, an immeasurable good would result to all. Any intelligent person would do well to try it on himself for a week, note the result and act accordingly. Many persons have fallen into the practice of drinking several glasses of cold water or several cups of hot tea at meals, out of mere habit ; all such will be greatly benefited by breaking it up at once ; it may be well to drink a little at each meal, and, perhaps, it will be found that in all cases it is better to take a single cup of hot tea at each meal than a glass of cold water, however pure.

